# Reaction to Ilesanmi OS et al. The managed hypertensive: the costs of blood pressure control in a Nigerian town

**DOI:** 10.11604/pamj.2013.14.85.2048

**Published:** 2013-03-05

**Authors:** Kingsley Nnanna Ukwaja, Cajetan C Onyedum

**Affiliations:** 1 Department of Internal Medicine, Federal Teaching Hospital, Abakaliki, Ebonyi State, Nigeria; 2 Department of Medicine, College of Medicine, University of Nigeria Teaching Hospital, P.M.B. 01129, Enugu Campus, Enugu, Nigeria

**Keywords:** Hypertensive, treatment, patient, financial, cost burden, Nigeria

## Reaction

We read with great interest the recently published article by Ilesanmi and colleagues in your journal [1]. The authors’ observation that patient's mean monthly cost of treatment for hypertension was ₦1440±560 ($9.6±3.7), equivalent to 11% of their mean monthly household income was very interesting. It made an interesting piece to read that patient illness costs due to care seeking for hypertension are largely undocumented in Africa just like has been observed for infectious diseases like tuberculosis [2].

The number of published studies that related patient costs of hypertension treatment in Nigeria is quite low despite the high burden of the disease [3]. There is therefore a need for a comprehensive data on the economic burden of hypertension care to patients and their households in the region in order to deliver appropriate interventions in Nigeria and indeed Africa.

In order to appropriately design future studies, we would like to present some further points that the study did not address. Although the authors highlighted in the background of their paper that direct costs constituted treatment-related costs and indirect costs constituted the lost man hours/savings among workers; it reported indirect costs as costs due to transportation which indeed should be part of the direct costs. Future studies should clearly define direct costs to include the out-of pocket (monetary) expenditures such as payments for administrative fees, drugs, laboratory tests, transport, hospitalization and food; while indirect costs defined as time loss of income such as transportation time, waiting times, absence from work. There is need to also document patients’ costs of coping with the hypertension issues like loans and, sale of assets [2].

About two-third 167 (66.8%) of the study participants were aged 60 years and above. This clearly indicates that the proportion having elevated blood pressure is more of the late middle age and elderly group. It also suggests that at this age they are more likely to visit the hospital with a family supporter (member). Such family supporters also incur additional direct (food, transportation) as well as indirect (waiting time, hospital visit, time due to transportation) costs: these needs to be considered in other to determine the full household costs of care per visit [2].

Household catastrophic expenditures for care have commonly been described as costs exceeding 10% of income [4]. Using this definition, the study showed that about 53% of the household of the patients incurred catastrophic expenditures. However, more recently, the World Health Organisation (WHO) have defined catastrophic expenditures as costs exceeding 40% of household income after basic food needs have been met [5]. It is recommended that in addition to determining average costs as a proportion of average income, the proportion of patient/households who incur catastrophic costs due to hypertension care according the current WHO definition should be assessed as findings using the new WHO definition may differ.

Despite being a monotherapy, alpha-methyl dopa emerged as the most cost effective drug in the cohort of patients studied. It is not clear if the stage of the hypertension determined the type of anti-hypertensive medication given to the patient. It is possible that patients with mild hypertension were more likely to receive monotherapy of alpha-methyl dopa which easily controlled the blood pressure. It is also possible that for patients who received alpha-methyl dopa-based antihypertensive combination therapy, the additional antihypertensives were introduced after a failure of the monotherapy. Previous studies have shown that drug class affected blood pressure'lowering efficacy in patients with differing severities of hypertension [6].

Post-diagnosis, Nigerian patients may require three to five re-treatments visits within 6 months to achieve target blood pressure levels [7]. The study did not mention the number of visits the patients had before they reached control. Thus, in order to appropriately perform a cost-effectiveness analysis of various drugs or combinations for hypertension treatment, there is need to consider the total costs of treatment from the number of re-treatment visits the patient has had before achieving control. It might have been better to present total costs per regimen required to achieve target blood pressure levels in terms of total patient /household costs per year.

Finally we would like to thank the authors for such an important study. We feel that their results should attract the attention of policy makers for better control of hypertension in Africa.
